# Metabolic targeting of EGFRvIII/PDK1 axis in temozolomide resistant glioblastoma

**DOI:** 10.18632/oncotarget.16767

**Published:** 2017-03-31

**Authors:** Kiran K. Velpula, Maheedhara R. Guda, Kamlesh Sahu, Jack Tuszynski, Swapna Asuthkar, Sarah E. Bach, Justin D. Lathia, Andrew J. Tsung

**Affiliations:** ^1^ Department of Cancer Biology and Pharmacology, University of Illinois College of Medicine at Peoria, Peoria, IL, USA; ^2^ Department of Neurosurgery, University of Illinois College of Medicine at Peoria, Peoria, IL, USA; ^3^ Department of Oncology, University of Alberta, Edmonton, AB, Canada; ^4^ Department of Pathology, University of Illinois College of Medicine at Peoria, Peoria, IL, USA; ^5^ Department of Cellular and Molecular medicine, Cleveland Clinic, Cleveland, OH, USA; ^6^ Illinois Neurological Institute, Peoria, IL, USA

**Keywords:** EGFR, EGFRvIII, DCA, glioblastoma, homology modeling

## Abstract

Glioblastomas are characterized by amplification of EGFR. Approximately half of tumors with EGFR over-expression also express a constitutively active ligand independent EGFR variant III (EGFRvIII). While current treatments emphasize surgery followed by radiation and chemotherapy with Temozolomide (TMZ), acquired chemoresistance is a universal feature of recurrent GBMs. To mimic the GBM resistant state, we generated an *in vitro* TMZ resistant model and demonstrated that dichloroacetate (DCA), a metabolic inhibitor of pyruvate dehydrogenase kinase 1 (PDK1), reverses the Warburg effect. Microarray analysis conducted on the TMZ resistant cells with their subsequent treatment with DCA revealed *PDK1* as its sole target. DCA treatment also induced mitochondrial membrane potential change and apoptosis as evidenced by JC-1 staining and electron microscopic studies. Computational homology modeling and docking studies confirmed DCA binding to EGFR, EGFRvIII and PDK1 with high affinity. In addition, expression of EGFRvIII was comparable to PDK1 when compared to EGFR in GBM surgical specimens supporting our *in silico* prediction data. Collectively our current study provides the first *in vitro* proof of concept that DCA reverses the Warburg effect in the setting of EGFRvIII positivity and TMZ resistance leading to GBM cytotoxicity, implicating cellular tyrosine kinase signaling in cancer cell metabolism.

## INTRODUCTION

Glioblastoma multiforme (GBM) is the most frequent and aggressive type of brain tumor. The median survival of patients remains only 12-15 months, despite innovations in neurosurgical techniques, development of chemotherapeutics and molecular targeted therapies [1]. The recent phase III randomized clinical trial performed by the European Organization for Research and Treatment of Cancer (EORTC) and the National Cancer Institute of Canada (NCIC) established surgery and regional radiotherapy with concomitant temozolomide (TMZ) daily during radiation therapy [2–4] as the current standard of care for newly diagnosed GBM. Although initial efficacy is high, more than 90% of GBMs recur and do not respond to TMZ, which is in part due to acquired TMZ chemoresistance [5]. While many approaches focus upon clarifying resistance, bypassing TMZ failure is a feasible alternative for developing additional strategies to regulate GBM growth [6].

GBM tumors are like most cancers in that they utilize aerobic glycolysis in the presence of adequate oxygen, which is referred to as the Warburg effect, although the advantage it confers in GBM cells remain unclear. However, the emergence of new potential targets for therapeutic intervention for molecularly targeted drugs has brought new promise that modulation of the Warburg metabolic phenotype could be targeted to further increase survival in GBMs. One intriguing candidate that has emerged is pyruvate dehydrogenase kinase 1(PDK1), reported to be important in promoting tumor metabolism and growth in variety of cancers including GBM [7–9]. Previously, we showed that PDK1 plays an important role in regulating GBM metabolism via simultaneous targeting of mitochondrial epidermal growth factor receptor (mtEGFR) protein levels and signaling activities with resultant tumor regression. PDK1 was selected for our study because of its involvement in the regulation of glucose metabolism by the TCA cycle. PDK1 phosphorylates the pyruvate dehydrogenase (PDH) E1α subunit and inactivates the PDH enzyme complex thereby preventing conversion of pyruvate to acetyl-coenzyme A, inhibiting pyruvate metabolism via the tricarboxylic acid (TCA) cycle [10]. In our previous results, we demonstrated that by targeting the ubiquitous EGFR found on GBM, DCA reversed this EGFR-mediated component of the Warburg effect by PDK1 binding ultimately reducing lactate production in GBM [9].

In continuation with our previous results and given the fact the EGFRvIII concurrently is overexpressed with EGFR and its expression is considered a hallmark for resistance to therapy [11, 12], we generated stable cells expressing EGFRvIII for further analysis. Additionally, we developed a cell line by continuous exposure of U373 cells constitutively expressing EGFRvIII to temozolomide (150μM) for 6 months. While it is known that treatment with TMZ is beneficial as a chemotherapeutic drug, GBM eventually progresses into acquired chemoresistance. This is the clinically relevant state to test potential novel targets for first line response. In this study we tested DCA targeting of mitochondrial EGFRvIII in TMZ-resistant glioma cells and xenografts. We demonstrated that DCA treatment increased cellular oxygen consumption, spared respiratory capacity and decreased lactate production in EGFRvIII expressing U373 and U373-TMZ resistant cells. Using xenograft experiments, we further showed that DCA treatment altered glucose metabolism by inhibiting the PDK1/EGFR/EGFRvIII interaction leading to mitochondrial membrane depolarization ultimately forcing the cells to undergo apoptosis.

## RESULTS

### PDK1 interacts preferentially with EGFRvIII

**Figure 1:**
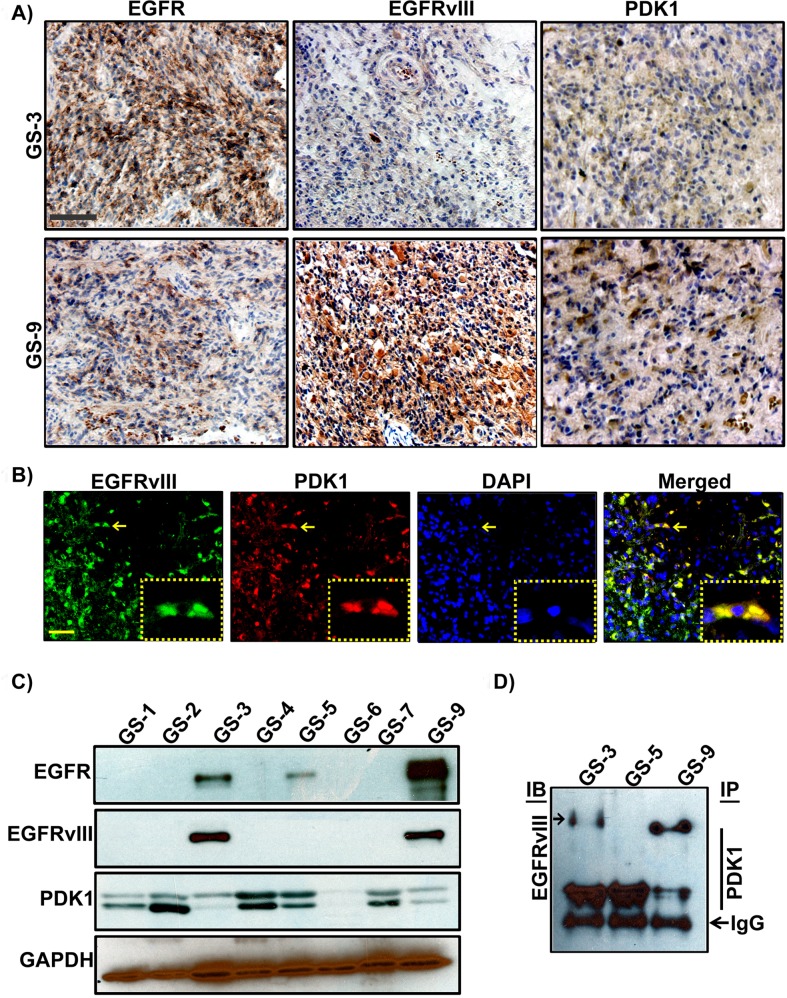
Detection of EGFR, EGFRvIII and PDK1 in hGBM clinical specimens **A**. Immunohistochemical staining of a primary human GBM with EGFR-, EGFRvIII-, or PDK1-specific antibody (top) on consecutive sections is shown (brown, diaminobenzidine; light blue, nuclear counterstain with DAPI; Bar=100 μm) **B**. Dual immunohistochemical staining for co-localization was conducted with anti-EGFRvIII and anti-PDK1 antibodies followed by the secondary antibodies conjugated with fluorophores for red (PDK1), green (EGFRvIII) and blue (DAPI) fluorescence, respectively. Representative merged images show the cells expressing PDK1 and EGFRvIII. Bar=100 μm. **C**. Western blot analysis of EGFR, EGFRvIII and PDK1 protein expression in hGBM specimens **D**. Immunoprecipitation experiments were conducted on hGBM patient specimen tissue lysates by using PDK1 antibody. Western blotting analysis was performed on these immuno-precipitated samples using EGFRvIII antibody. IgG probing was done to confirm equal loading.

To better understand how tyrosine kinase signaling in conjunction with PDK1 regulates the Warburg effect, we analyzed the expression of EGFR, PDK1 and EGFRvIII in different human surgical specimens (hGBM) using an immunohistochemistry-based approach. Previously we demonstrated that EGFR signaling contributes to GBM pathogenesis, and in this present report we examined the role of EGFRvIII in GBM tissues or cell line models. We used EGFR and EGFRvIII-specific antibodies (a gift from Celldex Therapeutics) to determine prevalence of the EGFRvIII and EGFRwt expression. EGFRvIII expression was observed to be more positive in tumors that also stained positive for EGFRwt expression. Interestingly, PDK1 expression positively correlated more with EGFRvIII than EGFR (Figure 1A). Immunohistochemical analysis conducted using Alexa Fluor antibodies confirmed EGFRvIII-PDK1 colocalization (Figure 1B). Immunoblots were done using lysates obtained from the corresponding tissues. Figure 1C shows wild type EGFR expression in three of eight representative tumor samples, while its mutant-EGFRvIII is detected in two. Immunoprecipitation experiments were further conducted with EGFRvIII positive samples (GS-3, GS-5 and GS-9) in western blot analysis. EGFRvIII interacted with PDK1 in GS-3 and GS-9 specimens only (Figure 1D).

### Computational approach confirms DCA binding to EGFRvIII, EGFR and PDK1

**Figure 2:**
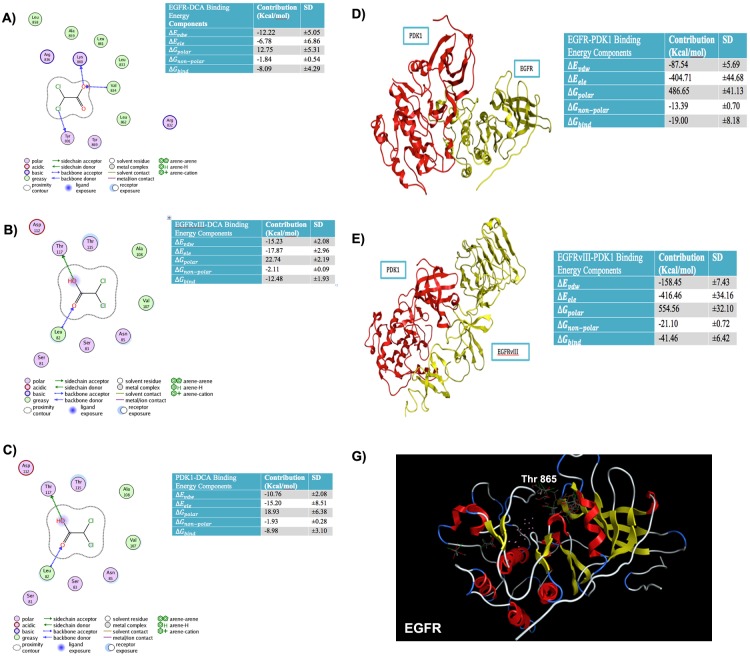
Binding energies and Protein Ligand Interaction fingerprint (PLIF) diagrams **A**. Binding Energies and Protein Ligand Interaction fingerprint (PLIF) diagrams showing atoms of DCA interacting with residues of **A**. EGFR **B**. EGFRvIII **C**. PDK1. Binding energies and structure of best pose for **D**. PDK1-EGFR complex **E**. PDK1-EGFRvIII complex. **F**. Top conformations of DCA docked in site close to TYR869 in case of wild type EGFR whereas this site does not anchor DCA conformations if TYR869 is mutated to Phenylalanine.

Earlier, we reported that DCA reduced EGFR phosphorylation at Tyr845, the essential site for mitochondrial translocation along with other EGFR phosphorylation [9]. Here, we explored the orientation and binding energies of DCA when docked with EGFR, EGFRvIII and PDK1. For this purpose, docking simulations were conducted using the molecular operating environment software (MOE, from the Chemical Computing Group, Canada). Orientation of DCA was explored using multiple DCA conformations. Figure 2A, shows PLIF (Protein Ligand Interaction Fingerprints) and binding energy components for the EGFR-DCA complex. PLIF is a 2-D representation of interactions of ligand (DCA) atoms with receptor residues. DCA was docked onto EGFR using a flexible docking microenvironment where side chains were allowed to move freely during placement of DCA. The structure of EGFR (PDB# 4WRG) was obtained from the PDB (protein data bank, www.rcsb.org). DCA is shown to interact with EGFR at residues, LYS860, VAL834 and TYR891 via hydrogen bonds. Alternatively, DCA also interacts with greasy residues like LEU858, ALA859, LEU861, LEU833, LEU862 and VALl834 hydrophobically. In another *in silico* experiment, we verified that DCA interacts with EGFRvIII electrostatically at THR117 and LEU82 and hydrophobically at LEU82, ALA108 and VAL107. It is important to note that the atomistic structure of EGFRvIII is not available within the protein data bank and so we created it by homology modeling (comparative modeling). We then extended our approach to further confirm the binding sites of DCA on PDK1. DCA binds to PDK1 (PDB#2XCH) at LYS111 electrostatically, at ASP223 using water-mediated hydrogen bonds and at LEU212, VAL96, LEU159, VAL143 and ALA109 hydrophobically. We additionally calculated the binding energies for EGFR-DCA, EGFRvIII-DCA, PDK1-DCA, PDK1-EGFR and PDK1-EGFRvIII complexes to be -8.09, -12.48, -8.98, -19.00 and -41.46 Kcal/mol, respectively (Figures 2A-2E). This indicates that DCA is likely to bind to EGFRvIII and to PDK1 with more favorable binding energies as compared to EGFR. Specific components of the binding energies shown in these tables are as follows: (a) Δ*E_vdw_*, representing the van der Waals contribution from molecular mechanism (MM) modeling; (b) Δ*E_ele_*, the electrostatic energy as calculated by the MM force field; (c), Δ*G_polar_*, the electrostatic contribution to the solvation free energy calculated by GB; (d) Δ*G_non-polar_*, the non-polar contribution to the solvation free energy calculated by an empirical model; and (e), Δ*G_Bind_*, the total estimated binding free energy calculated from the all the terms (a to e).

### Generation of temozolomide (TMZ) resistance model in constitutive EGFRvIII expressing GBM cells

**Figure 3:**
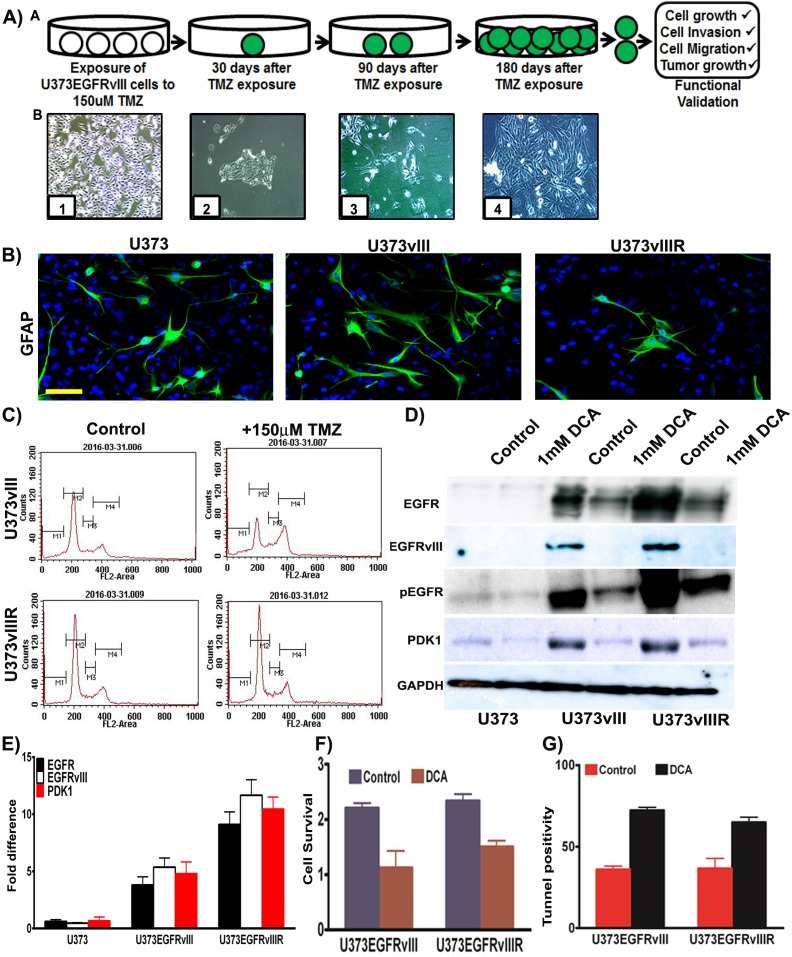
Development and validation of temozolomide resistant model **A**. Schematic representation depicting generation of U373vIII cells with 150μM TMZ resistance (U373vIIIR). Micrographs in lower panel demonstrate the timeline and development of U373vIIIR cells; Panel B1=confluent cells, B2=150 μM TMZ block, B3= TMZ resistant cells, B4=TMZ resistance cells growing to near confluence **B**. Immunofluorescence staining for the presence of GFAP was conducted on the U373, U373vIII and U373vIIIR cells followed by the secondary antibodies conjugated with green fluorophore. DAPI was used to stain the nuclei. Representative merged images show the cells expressing GFAP Bar=100 μm **C**. FACS analysis was done to analyze the cell cycle in U373vIII and U373vIIIR cells when treated with 150μM TMZ **D**. Western blot analysis of EGFR, EGFRvIII, pEGFR and PDK1 protein expression in U373, U373vIII and U373vIIIR cells. GAPDH was used as a loading control **E**. Semi-quantitative qRT-PCR analysis for EGFR, EGFRvIII and PDK1 in U373 and its EGFRvIII counterparts **F**. U373vIII or U373vIIIR cells were cultured with 1mM DCA and cell proliferation was measured by MTT assay **G**. Cells were stained for apoptosis using TUNEL assay. Results represent the mean ± SD of three experiments performed in triplicate.

GBM patients commonly exhibit resistance to TMZ treatment correlating with recurrence. TMZ resistant phenotype not only arises from methyl guanine methyltransferase (MGMT) [14], but also from the activation of different pro-survival pathways [15, 16]. Current approaches include modes of reversing TMZ resistance as well as anti-angiogenic control, cell surface antagonism via targeted therapy, and immunomodulation [17]. Our current effort has been devoted towards the implication of bioenergetics and oncogenesis as it pertains to the role of GBM recurrence. A barrier to the studies of GBM pathogenesis has been the availability of models that replicate the recurrent model in real time. To address this barrier, we developed a cell line by continuous exposure of U373 cells constitutively expressing EGFRvIII to temozolomide (150μM concentration) for 6 months with consideration of the respective EC50 values [18]. We selected and cultured the resistant surviving cell fraction of each passage and continuously re-exposured to TMZ after confluent growth (Figure 3A). These cells were tested for the expression of GFAP (Glial fibrillary acidic protein) and the immunofluorescence experiments confirmed its expression in U373, U373vIII and U373vIIIR cells (Figure 3B). The derived resistant cell lines are henceforth referred to as U373vIIIR. It is a known fact that TMZ induces cell cycle arrest and apoptosis [19] in glioma and here we conducted cell cycle analysis of cells expressing U373vIII and U373vIIIR and their treatment with 150μM TMZ using DNA flow cytometric analysis. After 48 h, U373vIII cells treated with 150μM TMZ decreased the number of cells in G1 from 53 to 34% and increased cells in S phase from 9 to 15% and in G2/M from 20.45 to 29% in line with other reported literature [19]. As expected, TMZ did not affect cell cycle profile and distribution in U373vIIIR cells (Figure 3C). Immunoblot analysis revealed increased expression of EGFR, EGFRvIII and PDK1 in the U373vIIIR cells compared to the U373vIII and U373 cells. Additionally, therapeutic response was visualized when treated with DCA (Figure 3D). Our immunoblot results were confirmed by RT-PCR analysis (Figure 3E). MTT analysis conducted to verify showed that 1mM DCA reduced the cell survival (about 50% in EGFRvIII expressing cells; 30% in EGFRvIIIR cells) (Figure 3F). Further, 1mM DCA treatment demonstrated increased apoptosis in EGFRvIII and EGFRvIIIR tunnel positive cells (Figure 3G). Collectively, DCA treatment demonstrated to be effective in regulating the U373vIII/U373vIIIR cell survival by the induction of apoptosis. We thus believe that the model we present here will serve as a clinical platform upon which data can be obtained in the setting of TMZ resistance.

### DCA decreases aerobic glycolysis in U373vIII/U373vIIIR cells; induces mitochondrial membrane potential (ΔΨm) and promotes mitochondrial apoptosis

**Figure 4:**
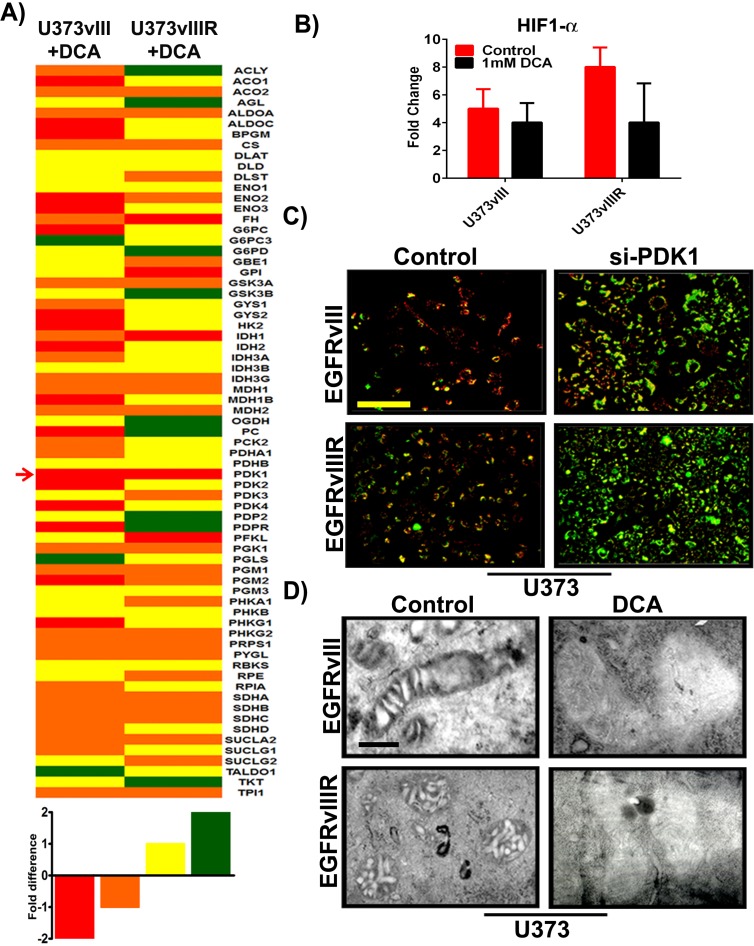
Differential expression of key genes involved in glucose metabolism; si-PDK1/DCA induce mitochondrial apoptosis and ΔΨm change **A**. Heat map demonstrating the differential expression of various key genes involved in GBM glucose metabolism. Red=strong downregulation, Orange=modest downregulation, Yellow=modest upregulation and Green=strong upregulation **B**. Quantification of *HIF1α* gene expression with response to 1mM DCA treatment on U373vIII/U373vIIIR cells (*n* = 4; *p* = 0.005). The data presented is normalized to loading control GAPDH **C**. GBM cells were transfected with siRNA for PDK1. After 72h of transfections, both control and treated cells were monitored for ΔΨm change using JC-1 dye and was analyzed by fluorescence microscopy (Red= J aggregation (live cells); Green=JC-1 monomer (dead cells) **D**. Identification of mitochondrial morphologies by electron microscopy. Bar=200 nm.

To gather a broad understanding of the effects of EGFRvIII overexpression and its TMZ resistance (EGFRvIIIR) on altered metabolism in glioblastoma, we used the human glucose metabolism RT2 profiler™ PCR array (cat # PAHS-006Z, SABiosciences, Valencia, CA) to study the expression of 84 key genes involved in the regulation and enzymatic pathways of glucose metabolism. Changes in glucose metabolic gene expression are a common feature of glioblastoma and specifically tumors show decreased oxidative phosphorylation, and reduced transcription of TCA cycle genes even in the presence of sufficient oxygen. Pharmacological inhibition of EGFR and PDK with DCA was reported recently to inhibit xenograft tumor growth in a variety of carcinoma and glioblastoma cancer cell lines [20, 21]. In the present study RNA from both the U373vIII and U373vIIIR along with their respective treatments using 1mM DCA were isolated and used for PCR microarray expression profiling. RNA expression profiles of U373vIII+DCA and U373vIIIR+DCA are depicted in heat-map format (Figure 4A). The genes listed were sorted based on overall expression levels in both conditions. In order to quickly scan if specific genes were differentially expressed in the U373vIIIR cells compared to the U373vIII cells, we used the simple ad hoc method fold change to calculate the EGFRvIII/ EGFRvIIIR expression ratio after DCA treatment. Genes with an expression ratio above -1.4 were considered highly downregulated, above -1.0 moderately downregulated and genes with an expression ratio of above 1.0 moderately upregulated, above 1.4 were considered highly upregulated. PDK1 was the sole gene highly downregulated in both cells tested suggesting that targeting PDK1 and EGFR in mechanisms that bypass TMZ resistance may be a key factor in reverting the Warburg aerobic glycolysis metabolic pattern in glioblastoma. Our previous results have shown that HIF1α regulates the PDK1/ EGFR interaction in the mitochondria and transcriptionally regulates PDK1 in glioblastoma [22, 23]. To further determine to what extent HIF1α regulates EGFRvIII/EGFRvIIIR and its interaction with PDK1 cells upon DCA treatment, we interrogated the expression of HIF1α. We validated our findings by qPCR and found that the genes encoding HIF1α were reproducibly lower in DCA-treated cells, when compared with that of their respective controls (Figure 4B). Taken together, our preliminary data show that DCA treatment in EGFRvIII/EGFRvIIIR GBM cells resulted in the coordinated downregulation of the expression of genes encoding members of glucose metabolism. We next investigated whether the induction of apoptosis in U373vIII/U373vIIIR cells by si-PDK1 involved alterations of mitochondrial membrane potential (ΔΨm), we examined its effect on ΔΨm in the aforementioned cells. Since induction of mitochondrial apoptosis mostly results in the loss of ΔΨm and measurement of ΔΨm is a sensitive measure for mitochondrial function *in vitro*, we examined the effect of si-PDK1 by JC-1 staining. U373vIII and U373vIIIR cells along with their aforementioned treatment were assessed at 24h post-treatment using the JC-1 dye. Fluorescence microscopy shows that control cells (untreated) had strong J-aggregation (red) and weak JC-1 monomer. In siPDK1-treatment, many cells showed strong JC-1 monomer (green staining) with concomitantly decreased J-aggregation (red staining) due to low ΔΨm (Figure 4C), thus indicating DCA induced ΔΨm change in GBM cells during apoptosis. To establish whether DCA induced cell death in GBM cells has the ultra-structural characteristics of apoptosis, mitochondria from U373vIII, U373vIIIR controls along with their DCA treated cells were compared using transmission electron microscopy (TEM). Mitochondria obtained from control cells showed vesicular and swollen structures indicative as a result of EGFRvIII overexpression as described previously [24]. Significant swelling and loss of mitochondrial membrane was observed in DCA treated cells, providing evidence that mitochondrial swelling may also be indicative of the apoptosis in GBM cells (Figure 4D).

### DCA reverses the Warburg effect in U373vIII/U373vIIIR cells

**Figure 5:**
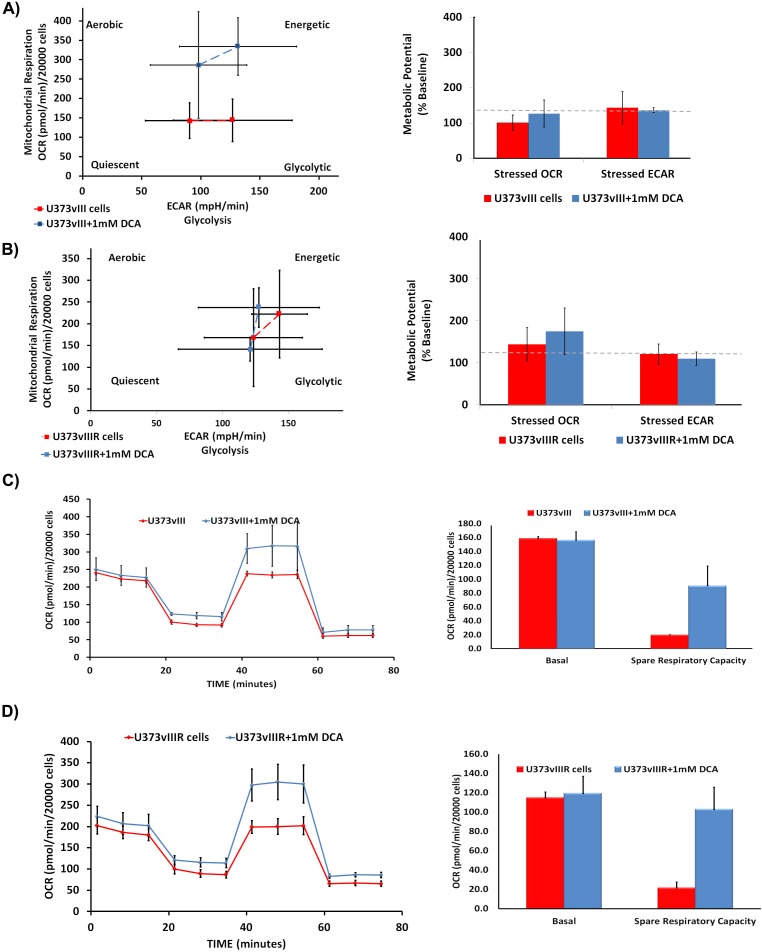
Measurement of bioenergetic parameters of U373vIII/U373vIIIR cells using Seahorse assays DCA treatment activates U373vIII **A**. U373vIIIR **B**. cells towards energetic phase. DCA treatment is believed to increase the aerobic potential as shown by the difference in stressed OCR between the control (blue) and treated (red) values. The assay was performed in triplicate. Effect of DCA on mitochondrial respiration and phenotype. U373vIII **C**. and U373vIIIR **D**. cells were treated with 1mM DCA 24 h. Oxygen consumption rate measured under basal conditions, following the addition of the oligomycin (mitochondrial F1-F0-ATPase inhibitor), FCCP (uncoupler) and rotenone (complex I inhibitor). Oxygen consumption rate (OCR) was measured using the Seahorse™ XFp Extracellular Flux analyzer. Each data point is the average of five independent measurements. Error bars indicate ±S.E.M.

We have previously demonstrated that DCA treatment reduced lactate production in EGFR overexpressing cells, suggesting the reversal of the Warburg effect. To gain mechanistic insights and to better understand if DCA plays a similar role in EGFRvIII overexpressing cells, we conducted cell energy phenotype assays using the Sea Horse Bioanalyzer. This assay delineates the phenotype of U373vIII/U373vIIIR under both baseline and DCA-stressed conditions. Oligomycin that inhibits ATP production was used at a concentration of 50 μM while FCCP (Carbonyl cyanide-4-(trifluoromethoxy) phenylhydrazone), a mitochondrial membrane depolarizer, was used at a concentration of 1μM (Figures 5A, 5B). U373vIII/U373vIIIR cells, when stressed with the aforementioned agents showed a glycolytic phenotype while DCA treatments showed the energetic phenotype. These results indicate that DCA treatment relieves U373vIII/U373vIIIR cells from ECAR (extracellular acidification rate) towards OXPHOS. Next, we queried the alterations of the major respiratory chain complexes in U373vIIIR cells compared to U373 cells. The cells were first treated with oligomycin, which decreases the OCR (oxygen consumption rate), and then were exposed to FCCP, which dissipates the mitochondrial membrane potential. The spare respiratory capacity is calculated as a measure of quantitative difference between maximal uncontrolled OCR and initial basal OCR. In this experiment, both U373vIII/U373vIIIR cells with and without DCA treatment were treated with DCA and exposed to the mitochondrial inhibitors rotenone and antimycin A. The DCA treatment groups showed increased spare respiratory capacities compared to the test controls (Figure 5C and 5D). These results suggest that DCA treatment may attenuate ECAR characterized by increased OCR, implicating that DCA treatment reverses the Warburg phenotype in cells overexpressing EGFRvIII and EGFRvIII cells resistant to TMZ.

### PDK1 expression correlates with EGFRvIII in mouse xenografts

**Figure 6:**
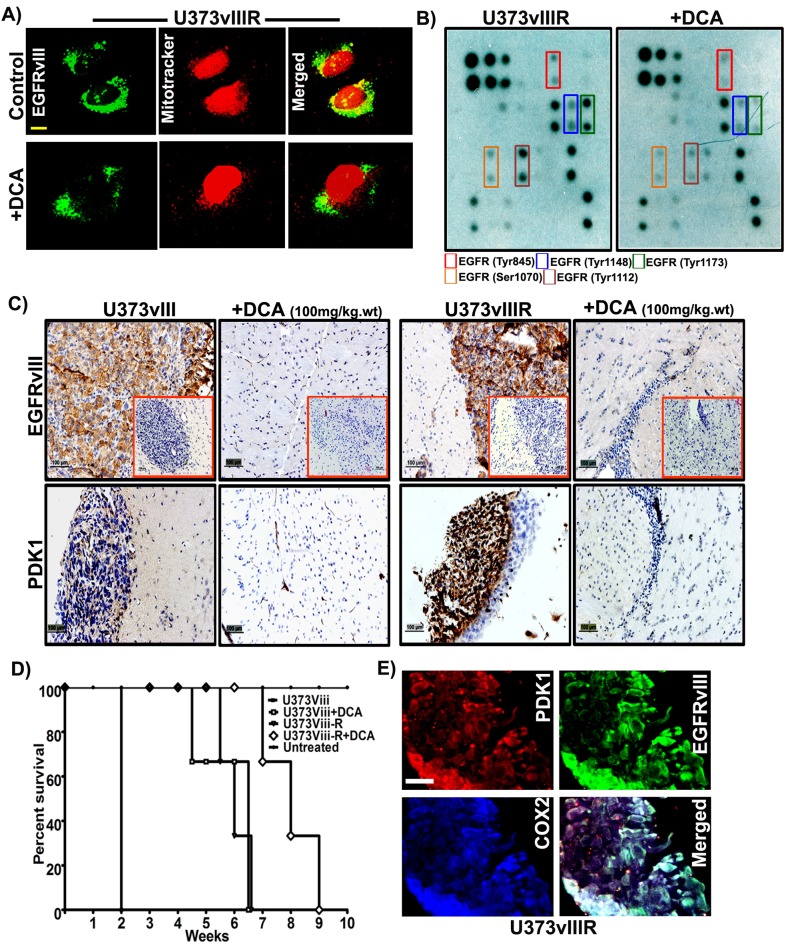
DCA treatment reduced EGFRvIII/EGFRvIIIR- induced tumor growth in mice **A**. Dual immunohistochemical staining for colocalization was conducted on the U373vIIIR cells with anti-EGFRvIII antibody and mitotracker followed by the secondary antibodies conjugated with fluorophores for green (EGFRvIII) and mitotracker (red) fluorescence, respectively. Representative merged images show the cells expressing EGFRvIII in colocalized in the mitochondria (Bar=100mm) **B**. Whole cell lysates of U373vIIIR and DCA treated cells were subjected to EGFR phosphorylation array and its representative images were presented **C**. Immuno-histochemical analysis of EGFRvIII and PDK1 expression in U373vIII/ U373vIIIR- induced tumors and their respective DCA treatment with 100gm/kg body weight. Representative H&E staining is seen in the insets of left panel (Bar=100mm) (*n* = 3) **D**. Kaplan–Meier survival curves from mice bearing intracranial EGFRvIII/EGFRvIIIR- induced tumors. DCA treatment group had an increased survival compared with the control group with a median survival 3-5 weeks. **E.** Immuno-fluorescent staining for PDK1 (red), EGFRvIII (green), COX2 (blue), demonstrating intense PDK1-EGFR co-localization (yellow) in U373vIIIR cells.

Having previously demonstrated that EGFR translocates to the mitochondria via Src based mechanisms, we hypothesized EGFRvIII also translocates to the mitochondria. To address this question, using confocal microscopy techniques, we examined the localization of EGFRvIII in U373vIIIR cells. EGFRvIII co-localized within the mitochondria in the control cells while DCA treatment nearly ablated all localization (Figure 6A). To gain mechanistic understanding into DCA binding to EGFRvIII, we conducted the EGFR phosphorylation array on the total protein lysates from U373vIIIR cells along with their respective treatment with 1mM DCA. Phosphorylation sites Tyr845, Tyr1148, Tyr1173, Ser1070 and Tyr1112 were observed to be increased in the U373vIIIR cells and DCA treatment reduced their expression (Figure 6B). Given the strong effect of DCA on down-regulating EGFRvIII/EGFRvIIIR/PDK1 on glioma cell lines, we investigated its effect in the mouse xenografts. We stereotactically implanted EGFRvIII/EGFRvIIIR cells intracerebrally into the right side of the brains of athymic nude mice in both control (U373vIII/U373vIIIR) and test (100mg/kg body weight) mice. DCA administered intravenously suppressed intracranial implanted EGFRvIII/EGFRvIIIR–induced tumor (insets of Figure 6C). To obtain more evidence of the relationship between PDK1/EGFRvIII/EGFRvIIIR, we analyzed paraffin-embedded tumors by immunohistochemistry (IHC). Mouse GBM xenografts tumor cells exhibited heterogeneous labeling of PDK1/EGFRvIII, with positive areas of staining detected alongside negative ones and the DCA treated tumors showed with very low PDK1/EGFRvIII (Figure 6C). Survival curves plotted revealed that DCA treatment increased the survival rate by more than 5 weeks in EGFRvIII treated mice and 3 weeks in EGFRvIIIR treated mice (Figure 6D). Immunocytochemistry experiments conducted on EGFRvIIIR cells revealed intense co-localization of PDK1 and EGFRvIII suggesting that PDK1 function is especially relevant for targeting the EGFRvIIIR-dependent GBMs (Figure 6E).

## DISCUSSION

Alterations in glucose metabolism of tumors have received considerable attention recently. Otto von Warburg reported that tumors rely on glycolysis rather than oxidative phosphorylation and proposed that tumor cells adopt oncogene driven metabolic reprograming to continue their deregulated proliferation, increased chemo-resistance and suppression of growth suppressors, a phenomenon termed the Warburg effect [25]. Most glial tumors demonstrate this characteristic upregulated glycolysis and are associated with weak mitochondrial activity [26] as evidenced by the high uptake of fluorodeoxyglucose (FDG) detected by Positron Emission Tomography (PET) technology [27]. Despite being the first line of therapy, GBM patients commonly exhibit resistance to TMZ treatment correlating with recurrence [28]. Chemoresistance is one of the main causes of failure yet the molecular mechanism in the setting of this phenomenon remains unclear [29]. Recently, Park et al showed that TMZ-induced a shift in pyruvate to lactate conversion preceding tumor suppression [30]. The expression of EGFR and particularly EGFRvIII is not only specific to GBM, but also implicit in pathological progression as well as recurrence. With EGFR mutations present in 50% and EGFRvIII alteration in 30-35% of GBM, inhibition of the tyrosine kinase signaling growth cascade has been proposed in numerous clinical trials [31].

GBM recurrence is heralded by an enriched fraction of EGFRvIII in the periventricular stem cell proliferating fraction, after radiation therapy, and after chemo and immunotherapy [32, 33]. Following this data, it appears although EGFR and EGFRvIII are undoubtedly characteristic and critical in pathogenesis, the downstream signaling and docking mechanisms have not been fully elucidated. With the initial report by Wallace et al showing EGFR shuttling to the mitochondria with a resultant Warburg phenotype, we focused upon the relationship of the key rate-limiting enzyme, PDK1 with EGFR and EGFRvIII as an alternate growth mechanism explaining the failure of cell surface targeting monoclonal antibodies and KLH vaccination strategies [34]. In this study, immunohistochemistry profiling corroborated the data initially presented by Heimberger revealing that EGFR is co-expressed with EGFRvIII [35]. We found a similar profile in our patients’ specimens.

Previously, we demonstrated mitochondrial EGFR promotes the Warburg effect by means of a positive feed-forward loop between PDK1 and HIF-1α. PDK1 phosphorylated and inactivated the mitochondrial pyruvate dehydrogenase complex, to serve as the link between ubiquitous cell surface signaling mechanism and distinctive GBM mitochondrial dysfunction. Recently Jutten et al. opined that EGFRvIII-expressing tumors required higher activation during metabolic stress [36]. On the other hand, Masui et al showed that EGFRvIII regulated glycolytic metabolism and its mediated tumor growth via Myc-dependent fashion [37]. Previously, we demonstrated that EGFR shuttles to the mitochondria as part of the metabolic modulation required for tumorigenesis and recently is shown to be involved in the expression of Glut1 and the Warburg effect [38]. To further explore the role of the Warburg effect in oncogenesis, we utilized dichloroacetate (DCA) to inhibit PDK1. DCA initially has been described in the treatment of lactic acidosis [39]. In recent years, others have found it to be active in cancer and specifically in GBM [40] While DCA was brought to the clinical setting in human trials in the treatment of GBM, it was met with failure since its safety was limited by peripheral neuropathy [41]. Our previous studies demonstrated that inhibition of PDK1 reverted the EGFR-induced Warburg effect away from glycolysis with resultant GBM cell death [9]. Postulating that failure may have less to do with specific metabolic targeting, and more upon the interaction of PDK with the ubiquitous EGFR altered signaling and docking mechanisms, we used *in silico* modeling methodology to test the critical binding sites of DCA. Computational models were created using molecular dynamic simulations of PDK1, EGFR and EGFRvIII with the goal of obtaining a high-confidence model of the interaction of DCA with each of the proteins. The docking site for DCA on PDK1 was identified using the experimentally determined structure defined by Kato et al. [42]. Docking simulations predicted the orientation of DCA in the binding pockets of EGFR, EGFRvIII and PDK1 and helped track the residues contributing to the binding. Binding energy calculations of protein-small molecule complexes show that DCA binds to EGFRvIII with higher affinity as compared to EGFR and PDK1. Further validation of EGFRvIII was shown with preferential binding of PDK1 to EGFRvIII compared to EGFR. Using the Kato et al. model we obtained binding energies for EGFR-DCA, EGFRvIII-DCA, PDK1-DCA, PDK1-EGFR and PDK1-EGFRvIII complexes and were shown to be -8.09, -12.48, -11.83, -52.69 and -55.08 kcal/mol, respectively. However, our current model provided better binding energies (Figure 2). Collectively, our modeling refinements to the Kato et al [42] reveal that the ultimate Δ*g* (total g-value) suggest that EGFRvIII-PDK1 is the most relevant interaction in cell surface mitochondrial interactions.

Although initial efficacy is high, acquired TMZ chemoresistance occurs in more than 90% of recurrent GBMs [43]. While many approaches focus upon clarifying resistance mechanisms, bypassing TMZ failure is a feasible alternative for developing additional strategies to regulate GBM growth [44]. The mitochondrial interaction with EGFR poses a uniquely targetable approach given three main factors: EGFR overexpression as a unique and specific marker in GBM, its implication in the pathogenesis and progression, and finally, the exceptional metabolic state induced by the Warburg effect [45]. We generated an *in vitro* model of TMZ dependent chemo resistance using U373 cell lines constitutively expressing EGFRvIII and our data showed DCA treatment showed selective response in the TMZ resistant U373vIII cells. We further verified that the actual bioenergetic status is altered correlating with upregulation of the above genes in the setting of TMZ resistance. We observed that mitochondrial electron transport increased in order to meet metabolic demand. This was evidenced in increased spare respiratory capacity (SRC) in DCA treated cells when compared to U373vIII and U373vIIIR controls. In addition, increased SRC mediated substrate entry to the TCA cycle correlated with increased electron transport chain activity shifting glycolysis to OXPHOS [46]. We show here, for the first time, that DCA treatment further increased SRC in U373vIII and U373vIIIR cells is suggestive of the fact that SRC is a product of co-regulated increase in the TCA cycle activity. Using a phenotype test kit, we observed that the U373vIII- and U373vIIIR cells show the glycolytic phenotype when stressed with Oligomycin and FCCP, with DCA treatment reverting towards the OXPHOS state. This phenotypic change results in decreased extracellular acidification in DCA treated cells suggesting a diminished requirement for glucose. This was not met with overall decrease in metabolism. Conversely, an actual increased basal and oxygen consumption rate was observed substantiating the concept of the reversal of the Warburg effect. This phenomenon could be the result of increased mitochondria or increased levels of enzymes of the electron transport complex. These results also point to the existence of correlative mechanism between TMZ resistance, altered metabolism and metabolic remodeling at the mitochondrial level. In conclusion, increased SRC and OXPHOS metabolism after DCA treatment, hypothetically indicate diversion from glycolysis towards the TCA cycle. Since this phenomenon is not clearly been demonstrated, this may represent a unique metabolic signature displayed after effective treatment and concomitant GBM cell death

Recently we and other groups demonstrated that EGFR, when phosphorylated at Y845 translocates to the mitochondria [47, 48]. Boerner et al further showed that phosphorylation of the c-terminal transmembrane domain (Y845 site) in EGFRvIII controls its translocation to the mitochondria and is independent of Src. This is one putative mechanism in chemotherapy resistance and failure of recent clinical trials [49]. In addition, EGFRvIII signaling protects the U87 cells from apoptosis by inducing expression of Bcl-X_L_ and enhanced lipogenesis [50]. Collectively, it can be inferred that while EGFRvIII is implicit in GBM progression, it's shuttling and docking to mitochondrial elements confers resistance by way of metabolic derangement. In this study, EGFRvIII showed increased mitochondrial translocation in the setting of TMZ resistance indicating its role in mediating cell metabolism while DCA treatment reduced its translocation. Furthermore, we observed that U373vIIIR cells showed increased phosphorylation at the novel residues Y1148, Y1173, S1070 and Y1112 in addition to Y845. Our data supports Warburg reversal in targeting the EGFR-PDK1 axis at the level of the mitochondria resulting in defined bioenergetic changes. Future therapies may thus be defined by taking into consideration characteristic cell surface markers and their pivotal role in docking and modulating the response to chemotherapy and immunotherapy, particularly in the resistant state so often encountered in glioblastoma.

## MATERIALS AND METHODS

### Reagents and plasmids

EGFR, pEGFR, PDK1, COX2, GFAP and GAPDH antibodies were purchased from Santa Cruz Biotechnology (Santa Cruz, CA). EGFRvIII antibody is a kind gift from Celldex Pharmaceuticals Inc. (Hampton, NJ). Temozolomide (TMZ) and PDK inhibitor dichloroacetate (DCA) were from Sigma (St. Louis, MO); *in* situ cell death detection kit was obtained from Roche (Indianapolis, IN). Human glucose metabolism PCR array (PAHS-006Z) was obtained from SABiosciences (Frederick, MD). JC-1 Mitochondrial membrane potential assay kit (Item No: 10009172) was obtained from Cayman Chemicals (Ann Arbor, MI). Mitotracker Red CMXRos (M7512) was purchased from ThermoFisher (Carlsbad, CA).

### Cell culture

U373 and U373vIII (EGFRvIII overexpressing) cells were a kind gift from Dr. Janusz Rak (Montreal Children's Hospital, Montreal, Canada). Both these cells were grown in DMEM high glucose media supplemented with 10% heat inactivated FBS, penicillin and streptomycin. Cells were incubated at 37°C in a humidified atmosphere containing 5% CO_2_. U373vIIIR cells were the resistant counter-parts of U373vIII cells that were subjected to 150μM temozolomide concentration over a period of 180days.

### Quantitative real-time PCR (qRT-PCR) and microarray analysis

The mRNA expression levels of EGFR, EGFRvIII and PDK1 were detected by qRT-PCR using the iCycler iQ (Bio-Rad; Hercules, CA) in the cDNA isolated from the naïve U373, U373vIII, U373vIIIR cells using the primer sequences listed in Supplementary Table 1. Each sample was run in triplicate for the target gene and the internal control gene GAPDH. In another experiment, we used human glucose metabolism RT2 Profiler PCR array (SA Biosciences, Frederick, MD) because of its advantage in detecting the expression of several genes concurrently. Each array contains a panel of 96 primer sets of 84 pathway-focused genes along with five housekeeping genes and three RNA and PCR quality controls. The RT-PCR was conducted as described by Velpula et al [13]. Changes in gene expression were illustrated as a fold increase/decrease with 1.0 or −1.0 as the cut-off. Genes that met these criteria were considered to be either up- or downregulated. All of these experiments were performed in triplicates.

### Docking studies for small molecule inhibitor-DCA

A flexible docking simulation was performed, where the binding site was assumed to be the entire protein. The side chains were kept free to move during force field refinement. Alpha PMI is the placement method used with default settings (sample per conformation = 10, maximum poses = 250). London dG rescoring was used with Alpha PMI placement. Termination criteria for force field refinement were set as gradient = 0.001 and iterations = 500.

### Molecular dynamics

Prior to docking all initial structures was energy-minimized using the molecular modeling force field of MOE. The leap module of Amber was used to add missing hydrogen atoms and heavy atoms using the Amber force field (ff99SB) parameters. To neutralize the charge of the system, we added an appropriate number of sodium ions. The model was immersed in a truncated cube-shaped shell of TIP3P water molecules. A time step of 2 fs and a direct-space, non-bonded cutoff of 10 Å were used. After the protein preparation, all systems were minimized to remove steric clashes. The systems were then gradually heated from 0 to 300 K over a period of 50 ps with constraints on solute, and then maintained in the isothermal–isobaric ensemble (NPT) at a target temperature of 300 K and pressure of 1 bar using a Langevin thermostat and Berendsen barostat, with a collision frequency of 2 ps and a pressure relaxation time of 1 ps, respectively. Hydrogen bonds were constrained using SHAKE. MD simulations were performed using the velocity-Verlet algorithm (default algorithm for the Amber MD package). The particle-mesh Ewald (PME) method was used to treat long-range electrostatic interactions using default parameters. Once the systems in our simulations reached the target temperature, they were equilibrated for 500 ps and the production run was continued for 40 ns in the isothermal–isobaric ensemble using the same Langevin thermostat and Berendsen barostat. Systems were simulated for a total of 40,600 picoseconds (ps). Out of this simulation time, 50 ps accounted for heating, 50 ps was density equilibration and 500 ps was equilibration at NPT, so that the system was simulated for 600 ps in addition to 40 ns of production run. Representative structures in the trajectories were collected at 10-ps intervals. The analysis of trajectories was performed with the PTRAJ module of Amber.

### Binding energy calculations

For the binding free energy calculations, we used the MM–GBSA method. We used MMPBSA.py python script. All water molecules and the sodium ions were excluded prior to MM–GBSA analysis. The values of the dielectric constant used for solute and surrounding water were 1 and 80, respectively. MM–GBSA analysis was conducted on 100 snapshots at the interval of 10 ps from the last 1ns of the 40 ns trajectory.

The final estimated binding energy was calculated using the following equation

Δ*G*_Bind_ = *G_Complex_* - *G_Receptor_*- *G_Ligand_* where *G* stands for free energy. The change in binding free energy is calculated as the sum of energies from molecular mechanics calculations, polar contribution and non-polar contribution to solvation free energy, i.e.

$$\Delta G_{\text{Bind}}=\Delta E_{MM}+\Delta G_{\text{pola}}+\Delta G_{\text{non}-\text{polar}}$$

In equation (2), *E_MM_* = *E_internal_* + *E_ele_* + *E_vdw_* · Δ*G_polar_* is the polar contribution to the solvation free energy and Δ*G_non-polar_* is the non-polar contribution to the solvation free energy, the latter defined as Δ*G_non-polar_* = Δ*SASA*+*b*, where *γ* = 0.0072 kcal mol^−1^ Å^−1^, *b*=0 for Amber GBSA calculations, and SASA is the solvent accessible surface area. The entropic energy *T*Δ*S* is normally subtracted from Δ*G_Bind_* in equation (2) but it is typically calculated by computationally expensive normal mode analysis; so we neglected entropic contributions to the binding free energy in our calculations. *E_MM_* is the molecular mechanics contribution to binding *in vacuo* expressed as the sum of the internal, electrostatic and van der Waals contributions. Since this is a single trajectory approach, the internal energy *E_internal_* will cancel out, so that *E_MM_* = *E_ele_* + *E_vdw_*.

### MTT and TUNEL assay

U373vIII/U373vIIIR cell growth was measured using the 3-(4, 5-dimethylthiazol-2-yl)-2, 5-diphenyl tetrazolium- bromide (MTT) assay. Cells were incubated with vehicle and DCA (1 mM), for 24h. Around 100μl MTT reagent (Invitrogen; Carlsbad, CA) was added to each well and the plates were incubated for 2h at 37°C to allow MTT to form formazan crystals by reacting with metabolically active cells. Medium was replaced with 300μl dimethylsulfoxide (DMSO) and plates were incubated for 30 minutes at room temperature with shaking. The optical density was measured at 595 nm. To evaluate cell death, TUNEL assay (Roche; Indiana polis, IN)was performed in both U373vIII and U373vIIIR cells treated with 1mM DCA following manufactures instructions.

### Western blot analysis, immunoprecipitation and immunofluorescence labeling

Proteins extracted from cultured cells and tissue lysates was followed by immunoprecipitation and immunoblotting with corresponding antibodies, as described previously [9]. Equal loading was confirmed by stripping and re-probing the membranes with GAPDH antibody. For immunofluorescence analysis, cells were fixed and incubated with primary antibodies, Alexa Fluor dye-conjugated secondary antibodies and DAPI (Molecular Probes) according to standard protocols. Cells were examined using Olympus BX61 confocal microscope with a 60-Å oil immersion objective. SPOT basic and SPOT advanced software was used to de-convolute Olympus BX61 images.

### Immunohistochemistry of hGBM specimens and *in vivo* tissue sections

hGBM surgical biopsy specimens were obtained from Saint Francis Medical Center, Peoria, IL and processed in accordance with the UICOMP Institutional Review Board–approved protocols. Serial sections of human specimens (GS-3 and GS-9) and mouse xenograft tissue sections were stained with the EGFR, EGFRvIII and PDK1 antibodies as described previously [9]. Images were acquired by using an Olympus BX61 fluorescence microscope and processed using SPOT advance software (Diagnostic Instruments; MI).

### Transmission electron microscopic studies

The cells were fixed using fixative solution (2.5% glutaraldehyde in 0.1 M phosphate buffer, pH 7.4). After fixation, samples were buffer rinsed and post fixed with 1% osmium tetroxide, dehydrated (35%, 70%, 95%, 100% ethanol dehydration), and flat embedded in propylene oxide and Epon 812 epoxy resin (Tousimis) in 1:1 ratio at 60°C for 4 h. A Reichert OMU3 ultramicrotome (Austria) was used to prepare 700A° thin sections that were mounted on 200 copper mesh grids, stained with uranyl acetate and lead citrate. The sections were viewed under a JEOL JEM 100C transmission electron microscope (60 kV).

### Mitochondrial permeability potential

Both U373vIII/U373vIIIR cells along with their counterpart treatments with 1mM DCA were stained with the cationic dye JC-1 (Cayman Chemicals (Ann Arbor, MI), which exhibits potential-dependent accumulation in mitochondria. At low membrane potentials, JC-1 continues to exist as a monomer and produces a green fluorescence (emission at 527 nm). At high membrane potentials or concentrations, JC-1 forms J aggregates (emission at 590 nm) and produces a red fluorescence.

### Cellular bioenergetics analysis using XFp extracellular flux analyzer (seahorse biosciences)

Both U373vIII/U373vIIIR cells were plated at a density of 30,000/well in an XFp eight well plate. Cells were allowed to grow overnight in 1mM DCA and the media were exchanged 1 hour before the assay with XFp media to determine the oxygen consumption rate (OCR) and extracellular acidification rate (ECAR). For cell energy phenotype assay, FCCP and oligomycin were diluted into XFp media and loaded into the accompanying cartridge to achieve final concentrations of 10μM; for mitostress assay, oligomycin, FCCP and rotenone/antimycin A were diluted to achieve final concentrations of 1.0μM and 0.5μM respectively. Injections of the drugs into the medium occurred at the time points specified. These experiments were done to define a basal OCR, ATP-linked OCR, proton leak, maximal respiratory capacity, reserve respiratory capacity, and non-mitochondrial oxygen consumption. All reagents were purchased from Seahorse Biosciences. Results were representative of at least 3 independent experiments.

### EGFR phosphorylation antibody array

Around 500 μg of total cell lysate from the U373vIIIR cells and their treatment with 1 mM DCA were subjected to EGFR phosphorylation antibody array (Ray Biotech, Norcross, GA) to detect the phosphorylation status of EGFR in DCA treatments. The assays were performed as described previously [9].

### Intracranial injections

Around 1×10^6^ U373vIII or U373vIIIR cells were intracranially injected into 4-week-old female athymic nude mice (female 6–8-week-old, Harlan Labs), by following the standard protocol [9]. Four mice per group were used in each experiment. Two weeks after tumor implantation, the mice were intravenously injected with 100 mg/kg body weight of DCA. Tumor formation and the phenotype were determined by histologic analysis of hematoxylin and eosin stained sections.

## SUPPLEMENTARY TABLE


