# Supplementary material for: Metabolic targeting of EGFRvIII/PDK1 axis in temozolomide resistant glioblastoma
Source: Oncotarget. 2017 Mar 31;8(22):35639–55. doi: 10.18632/oncotarget.16767 (PMC5482605; doi:10.18632/oncotarget.16767)
Supplement: Supplementary file 1 [file oncotarget-08-35639-s001.pdf]

Metabolic targeting of EGFRvIII/PDK1 axis in temozolomide resistant glioblastoma

Supplementary Material

Supplementary Table-1

EGFR-F:	CCACCAAATTAGCCTGGACA
EGFR-R:	CGCGACCCTTAGGTATTCTG
EGFRvIII-F:	CTCTGGAGGAAAAGAAAGGTAA
EGFRvIII-R:	AGGCCCTTCGCACTTCTTAC
PDK1-F	CTGTGATACGGATCAGAAACCG
PDK1-R	TCCACCAAACAATAAAGAGTGCT
GAPDH-F	AATCCCATCACCATCTTCCA
GAPDH-R	TGGACTCCACGACGTACTCA
